# Curcumin Internalization in *Streptococcus mutans* Biofilms: A Confocal Microscopy Analysis

**DOI:** 10.1002/jbio.202500357

**Published:** 2025-08-13

**Authors:** Rebeca Vieira de Lima, Bruno Pereira de Oliveira, Francisco Eduardo Gontijo Guimarães, Kate Cristina Blanco, Vanderlei Salvador Bagnato

**Affiliations:** ^1^ Biotechnology Postgraduate Program (PPG Biotec), Federal University of São Carlos São Carlos São Paulo Brazil; ^2^ São Carlos Institute of Physics (IFSC), University of São Paulo (USP) São Carlos São Paulo Brazil; ^3^ Department of Biomedical Engineering Texas A&M University College Station Texas USA

**Keywords:** biofilm, confocal microscopy, oral health, photosensitizer, *Streptococcus mutans*

## Abstract

*Streptococcus mutans* is one of the main harmful agents to oral health, exhibiting high resistance in its biofilm form. This study evaluated curcumin as a photosensitizer in photodynamic inactivation (PDI), monitoring its internalization time and activity. The biofilm was cultured for 24 h and treated with curcumin activated by two‐photon excitation (800 nm). After photodegradation, curcumin continued to penetrate effectively into the biofilms, replacing previously degraded molecules with new ones and constantly generating reactive species (ROS and singlet oxygen) capable of damaging the bacteria. This contrasts with previous studies that reported limitations of natural photosensitizers in this context. Therefore, the principal contribution of this study is the in vitro demonstration of the dynamic efficacy of curcumin in the complex biofilm environment. The use of confocal microscopy was essential to visualize and quantify the effects of curcumin, highlighting its value as an analytical tool in the evaluation of biofilm treatments.

## Graphical abstract

*Streptococcus mutans* are harmful bacteria to oral health, exhibiting high resistance in their biofilm form. This study evaluated, through Confocal Microscopy, curcumin extracted from *Curcuma longa* as a photosensitizer in photodynamic inactivation (PDI), monitoring its internalization time and activity. The study demonstrated, in vitro, the dynamic efficacy of curcumin within the complex biofilm environment.

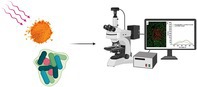

## 1 Introduction

**FIGURE 1:**
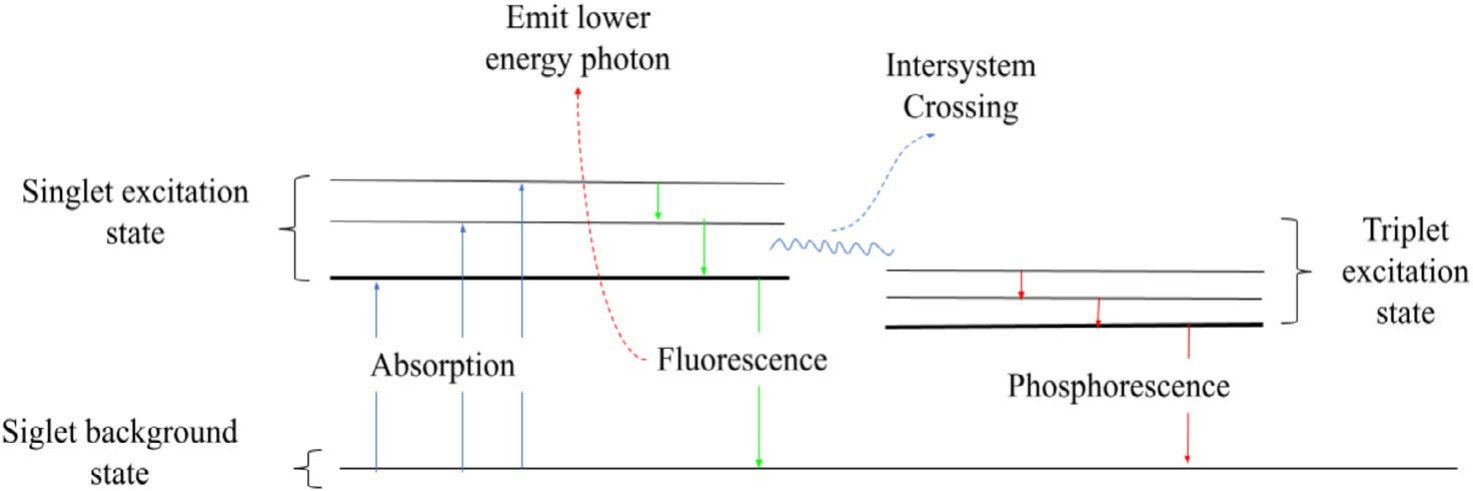
Jablonski diagram illustrating the process of luminescence. Upon absorption of light (blue arrows), an electron is excited from the singlet ground state to a higher energy level in the singlet excited state (Fluorescence) and then undergoes intersystem crossing to an excitation level in the triplet (phosphorescence). After a brief period, the electron returns to a lower energy level by emitting a photon with lower energy, resulting in fluorescence (green arrows). This emitted photon corresponds to the luminescence observed in the process. The energy difference between absorption and emission causes the emitted light to have a longer wavelength (lower energy) than the absorbed light.

The oral microbiota, particularly Gram‐positive bacteria such as *Streptococcus mutans* [1, 2, 3], plays a crucial role in the occurrence and progression of oral diseases, including dental caries, periodontal disease, and oral cancer [4]. Additionally, dysbiosis of the oral microbiota is associated with various systemic diseases, such as inflammatory bowel disease, respiratory conditions, and diabetes mellitus [5, 6, 7, 8]. Biofilm formation occurs when bacteria adhere to solid surfaces, such as teeth, and produce an extracellular polysaccharide matrix, creating a structured microbial community [9, 10]. Biofilm is critical for bacterial resistance as it protects the bacteria within, shielding them from environmental challenges and antimicrobial agents. This protection promotes physiological and structural changes that enhance virulence, persistence, and resistance to antimicrobials [11, 12, 13, 14]. Photodynamic inactivation (PDI) has emerged as a promising alternative for treating localized infections, particularly in targeting multidrug‐resistant bacteria, without promoting resistance to photosensitizers (PS) [15, 16]. However, the effectiveness of PDI on microorganisms protected by biofilms remains limited due to the biofilm acting as both a physical and chemical barrier that reduces PS penetration and efficacy [17]. This represents a challenge in dental clinics, where PS such as methylene blue and toluidine blue are commonly used, but often result in tooth discoloration, unlike curcumin, which, despite its strong yellow color, has the advantage of not causing staining [18, 19]. The interaction between biofilms and PDI is complex, as the extracellular matrix of biofilms acts as a shield, impeding PS penetration and reducing their effectiveness. However, the use of curcumin as a photosensitizer presents several advantages in this context. In addition to not causing tooth discoloration, curcumin exhibits ideal properties, such as absorption in the blue light region, high triplet quantum yield, and the ability to penetrate cell membranes [20]. Therefore, it is necessary to explore the efficiency of curcumin, in combination with lasers, for the deconstruction of *S. mutans* biofilms. Given this background, this study hypothesizes that curcumin, when used as a photosensitizer in PDI, promotes significant inactivation and deconstruction of *S. mutans* biofilms due to its ability to penetrate biofilm layers homogeneously and its effective interaction with blue light. Additionally, we hypothesize that curcumin will demonstrate higher penetration and efficacy in biofilms than in planktonic forms. To test these hypotheses, confocal microscopy was employed to visualize and quantify curcumin internalization and its ability to promote photoinactivation of biofilms. The process of phosphorescence can be visualized using a Jablonski diagram (Figure 1), where the absorption of light excites an electron from the singlet ground state (S_0_) to a higher energy level in the singlet excited state (S_1_) and the triplet excited state. This conjecture is a luminescence principle. After a short period, the electron returns to a lower energy level, emitting a photon with lower energy in the process, known as phosphorescence. This emitted photon has a longer wavelength (lower energy) compared to the absorbed photon due to the energy difference between the excited and ground states.

**FIGURE 2:**
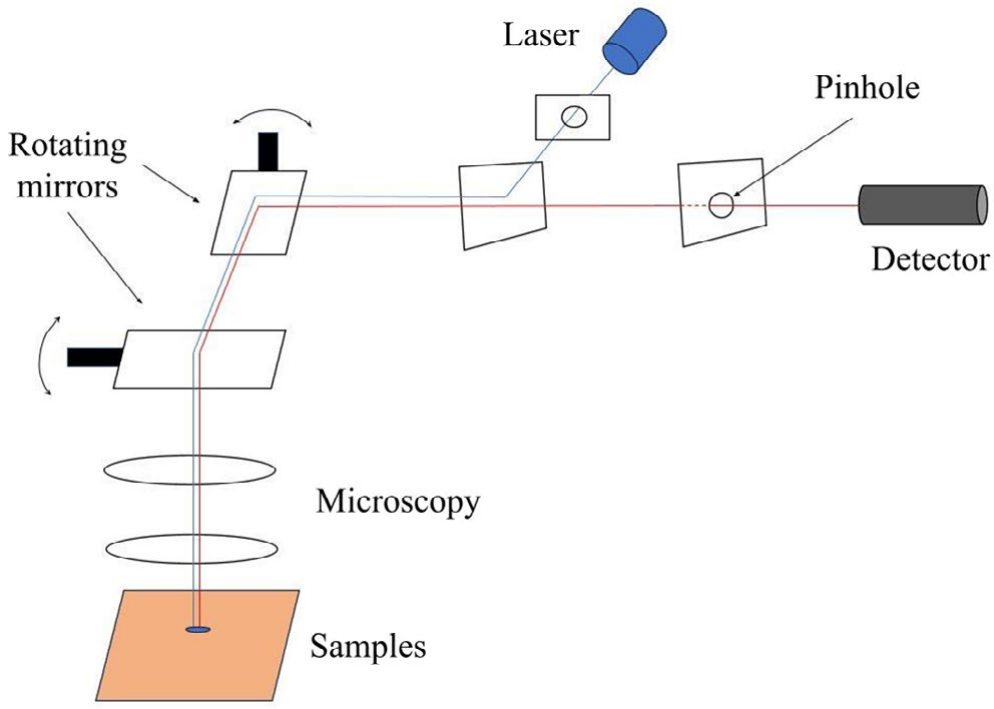
Schematic representation of a confocal microscopy setup. The laser source emits a light beam, which is directed by a series of rotating mirrors toward the sample. The beam passes through the optical lenses of the microscope, focusing on the sample. Luminescent light emitted from the sample is collected and directed back through the optical system. A pinhole is placed before the detector to block out‐of‐focus light, ensuring that only the light from the focal plane reaches the detector. This allows for high‐resolution imaging of the sample. The detector captures the luminescence signal, which is processed to create detailed images of the sample.

Luminescence spectroscopy is widely applied in biological experiments, particularly with recent advancements in optical instrumentation, which enable the characterization of molecular profiles and their interactions [21, 22]. Curcumin, as a fluorescent molecule, can be excited by blue light, emitting light of different colors as it returns to its ground state, allowing for visualization and analysis via confocal microscopy [23]. Confocal microscopy excels in constructing high‐resolution three‐dimensional images, enabling detailed analysis of curcumin internalization within bacterial biofilms, which is crucial for the success of PDI [24, 25]. A confocal microscopy setup (Figure 2) involves a laser source that emits a light beam, which is directed by rotating mirrors toward the sample. The beam passes through the microscope's optical lenses, focusing precisely onto the sample. Luminescent light emitted from the sample is collected and sent back through the optical path. A pinhole is placed before the detector to block any out‐of‐focus light, allowing only the luminescence from the focal plane to reach the detector. This setup ensures high‐resolution imaging, and the detector captures the luminescence signal, which is processed to generate detailed images of the sample.

## 2 Materials and Methods

### 2.1 Preparation of Curcumin Solution

**FIGURE 3:**
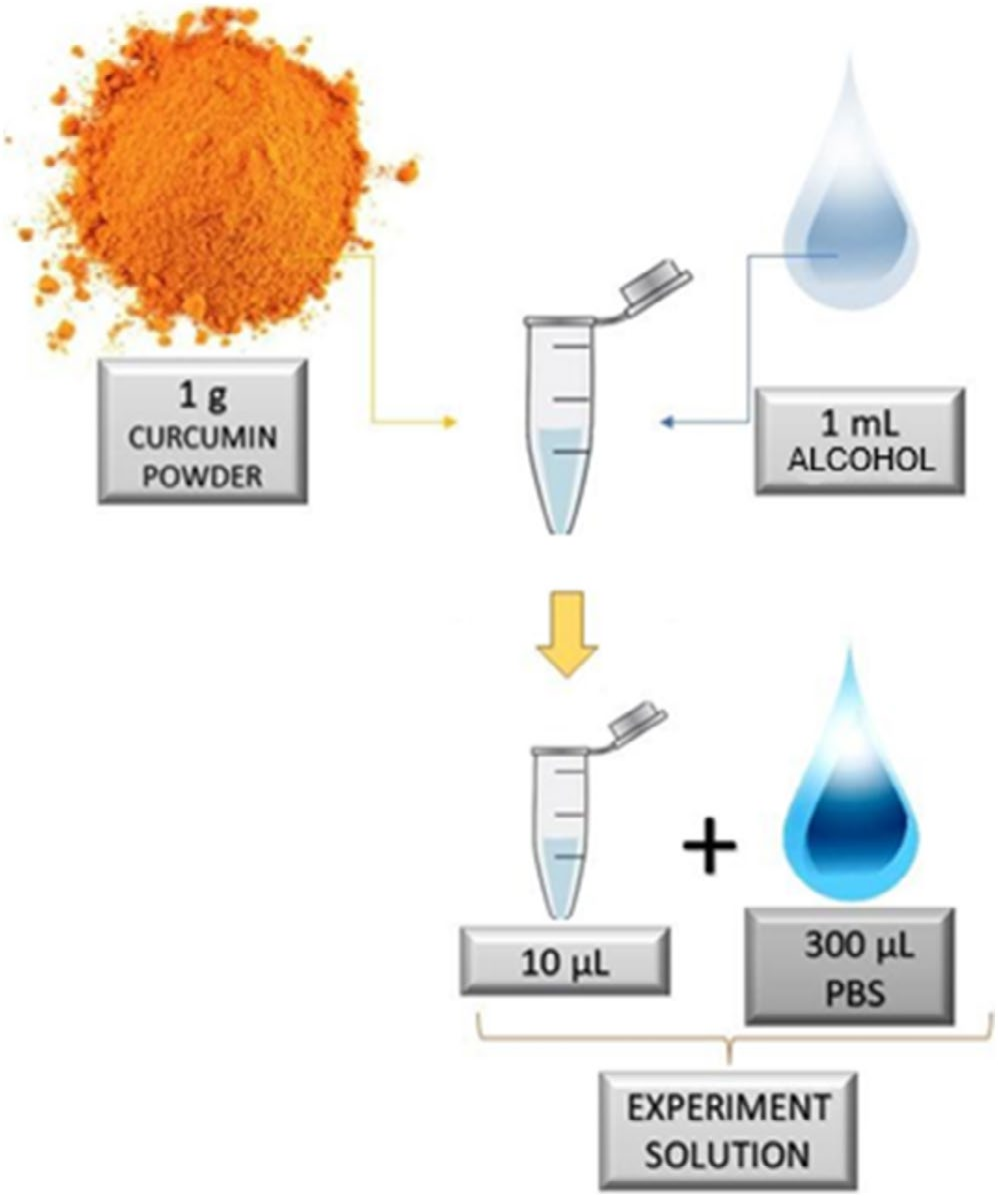
Preparation of experimental curcumin solution: 1 g of curcumin powder is dissolved in 1 mL of ethanol, from which 10 μL is diluted in 300 μL of PBS to create the experimental solution.

Curcumin is insoluble in water, so a stock solution of photosensitizer was prepared from 1 g of curcumin powder dissolved in 1 mL of absolute alcohol P.A. ACS (99.5%). To prepare the solution used in experiments, 10 μL taken from the stock solution was diluted in 300 μL of phosphate‐buffered saline (PBS). Each step is visually represented by arrows, indicating a sequential preparation process for the final solution (Figure 3).

### 2.2 Bacterial Culture Conditions and Inoculum Preparation

Strain *Streptococcus mutans* ATCC 25175 is kept in cryogenic vials under refrigeration at −4°C in Brain Heart Infusion (BHI) culture broth supplemented with 20% glycerol. To prepare the inoculum, samples were inoculated into falcon tubes with 20 mL of BHI culture medium containing 1% sucrose and placed on an orbital shaker at 37°C overnight. After incubation, 50% of the planktonic culture was washed twice with phosphate buffered saline (PBS) to remove non‐adherent bacterial cells. The inoculum was adjusted to 10^8^ CFU/mL at 600 nm using a Cary UV‐Vis50 spectrophotometer (Varian).

### 2.3 Biofilm Formation

Biofilms were formed in the wells of Cell view cell culture plates with four sections by transferring 1 mL of activated inoculum to each well section. The plates were incubated in a Biochemical Oxygen Demand (BOD) incubator at 37°C (±2°C) for 24 h. After biofilm formation, the supernatant was carefully removed, and the biofilms were washed twice with PBS to remove any planktonic cells. The biofilm thickness and homogeneity were controlled by confocal microscopy, and each biofilm was treated and analyzed in independent wells to ensure consistency. For each experimental condition, biofilms were grown in triplicate, with at least three independent experimental replicates carried out to ensure reproducibility. Any variability in biofilm formation was minimized by standardizing growth conditions, such as inoculum size, incubation time, and temperature. In addition, statistical analysis was carried out using ANOVA to assess the significance of differences between treated and control groups, ensuring a robust interpretation of results.

### 2.4 Confocal Microscopy Analysis

Curcumin at a concentration of 10 μg/mL (PDT Pharma LTDA) was added to the biofilm samples, and these were transferred to the confocal microscopy chamber. Confocal microscopy analysis was carried out using light doses of 0.1 J/cm^2^ per pixel to minimize photodegradation during imaging, and additional experiments were also carried out with higher doses (30 J/cm^2^) for comparison. Activation of the photosensitizer molecule was done through excitation with two photons at a wavelength of 800 nm. This process, also known as Two‐Photon Absorption (TPA), consists of an atom or molecule absorbing two photons simultaneously instead of one to be excited to a higher energy state. To validate the results of PDI, positive and negative controls were included in the experimental design. For the negative control, biofilms and planktonic cells were treated with curcumin without exposure to light, ensuring that effects observed were due to PDI and not curcumin on its own. For the positive control, biofilms were treated with light exposure in the absence of curcumin to confirm that photoinactivation effects were specific to the photosensitizer. In each condition, bacterial viability was assessed using Live & Dead dye (Sigma‐Aldrich), with ethidium bromide indicating cell damage (red fluorescence)—as it only stains the cell that has suffered damage in the cellular wall—and acridine orange indicating viable cells (fluorescence). Additionally, confocal microscopy was used to monitor curcumin incorporation into biofilm and planktonic cells by applying the photobleaching technique. Biofilms were treated with 10 μL of curcumin solution, and images were captured to assess both the incorporation of curcumin and the viability of cells after treatment. Fluorescence recovery after photobleaching (FRAP) was performed to quantify curcumin retention and photodegradation in both biofilm and planktonic forms. By implementing these experimental controls and replicating assays, we ensured the reliability of PDI results, providing a robust comparison between treated and untreated biofilms and between biofilm and planktonic forms. Statistical analyses were performed to determine the significance of observed effects.

## 3 Results

**FIGURE 4:**
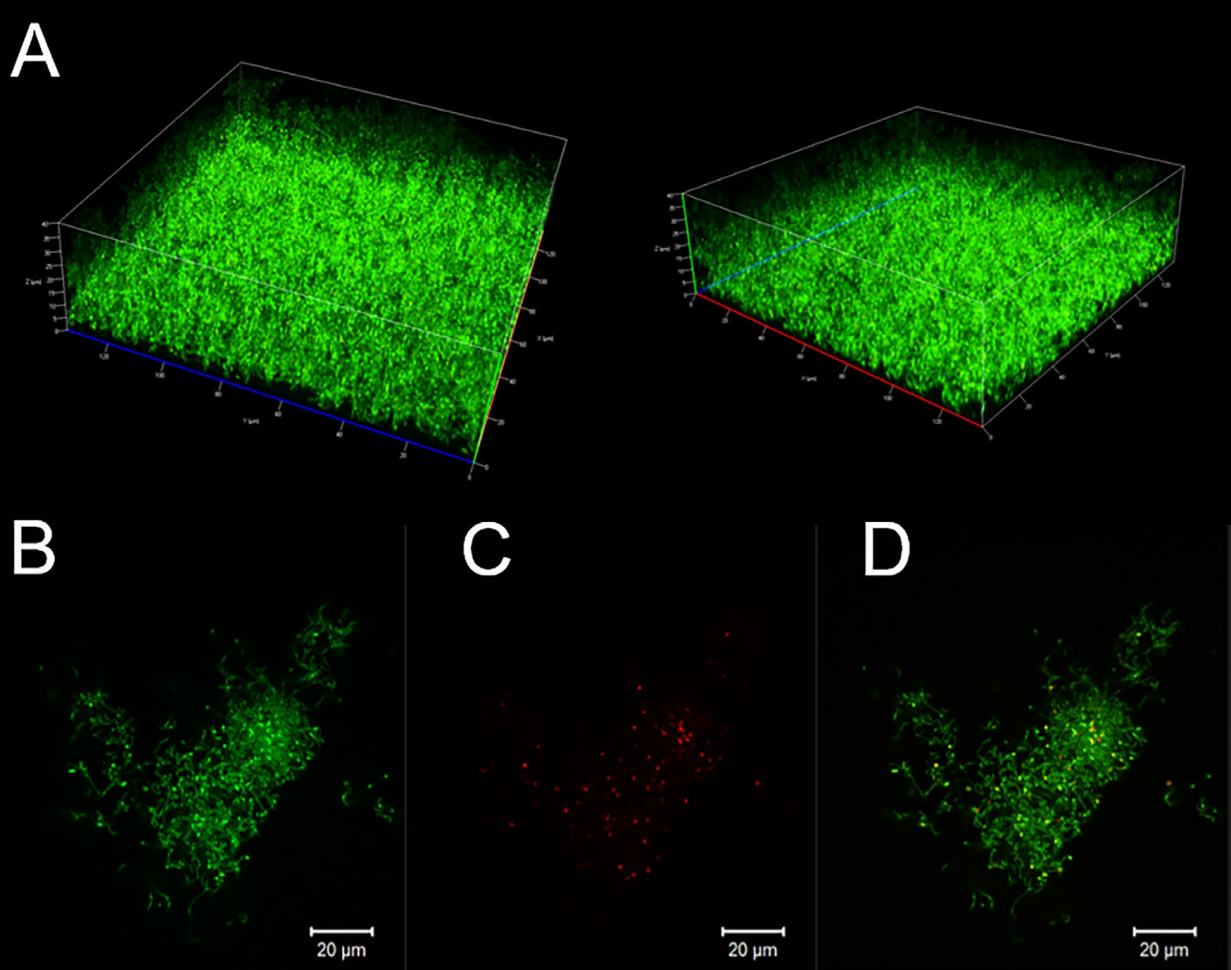
(A) A 3D confocal microscopy image of *S. mutans* biofilm, showing a homogeneous structure with an approximate thickness of 40 μm. (B) *S. mutans* biofilm viability test using Live & Dead dye. Green fluorescence indicates viable bacteria. (C) Red fluorescence marks non‐viable cells within the biofilm. (D) An overlay image of the viable (green) and non‐viable (red) bacteria, highlighting the distribution of live and damaged cells within the biofilm structure. Scale bars in B–D represent 20 μm.

Microbial activity in biofilms was determined using confocal microscopy that excited in through by two photons in 800 nm and constructed the 3D control image to obtain in channel mode at a confocal plane near the glass slide—1.6 μm deep—containing analyzed culture (Figure 4A). Firstly, we can demonstrate biofilms were homogeneous with an average thickness of 40 μm (±5 μm). Second, results are in Figure 4B, which showed the biofilm stained with Live & Dead dye, and the confocal microscopy image shows predominantly green fluorescence, indicating most bacterial cells in a biofilm are viable. Consequently, we have Figure 4C, which shows the same biofilm as Live & Dead dye, but with red fluorescence, which corresponds to non‐viable or damaged bacterial cells inside the biofilm. Figure 4D shows a full image of green and red fluorescence from Figure 4B,C together, showing live (green) and damaged (red) bacterial cells in the biofilm. This combination allows clear visualization of viable and non‐viable cell distribution in the biofilm. For all the images in this manuscript, scales of 20 μm were standardized.

**FIGURE 5:**
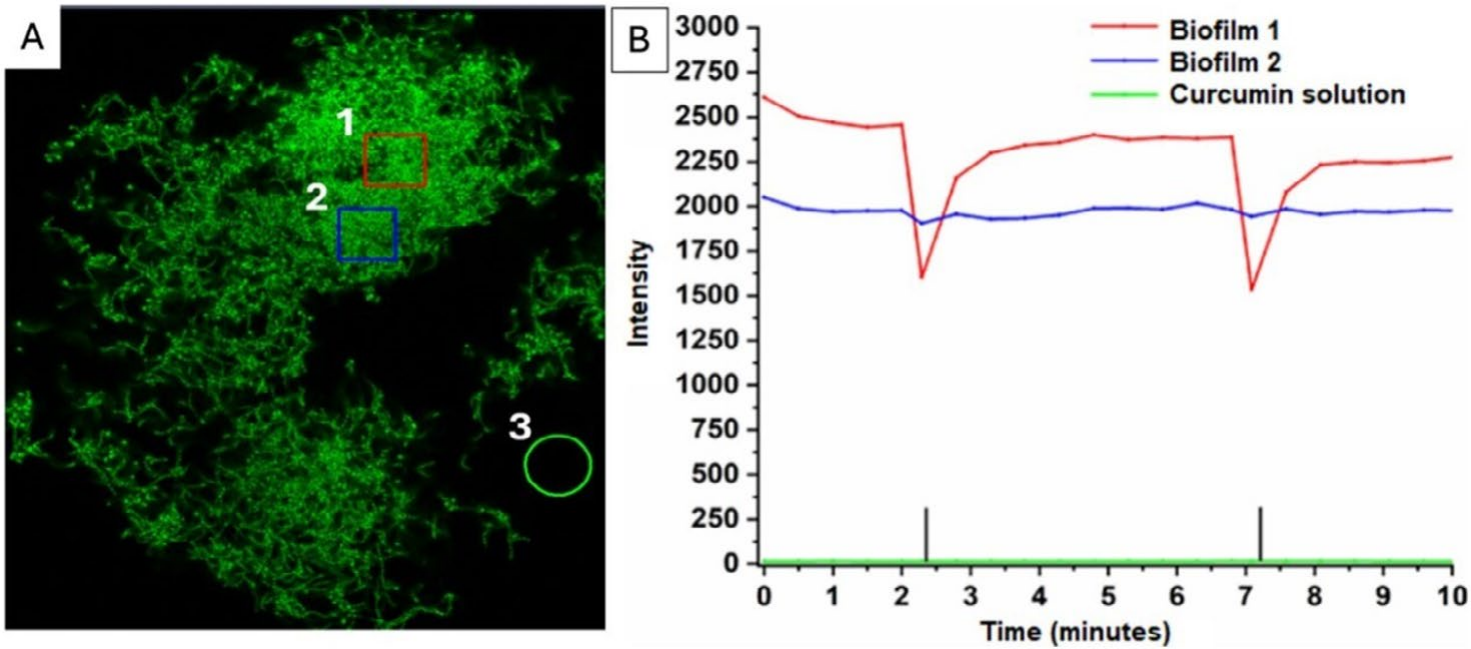
(A) Confocal microscopy image of *S. mutans* biofilm showing three selected areas for analysis: Area 1 (red square) underwent two photodegradations; Area 2 (blue square) only incorporated curcumin solution without photodegradation; Area 3 (green circle) represents the curcumin solution in the medium. (B) Graph showing intensity changes over 10 min. Vertical black bars indicate the timing of photodegradation events. 20 μm scale.

*S. mutans* biofilms and planktonic forms were exposed to a light dose of 30 J/cm^2^. For an initial analysis, three regions were marked to verify fluorescence intensity over time. Biofilm area 1 (Figure 5A—red square) shows the highest initial intensity but decreases after photodegradation and recovers over time—in approximately 2–3 min—(Figure 5B—red line). Biofilm area 2 (blue square) corresponds to untreated biofilm, whose intensity remains constant without photodegradation (Graph 5B–blue line) and free curcumin solution in medium (green circle) without exposure to light remains constant, that is, does not show significant fluorescence (Graph 5B–green line).

**FIGURE 6:**
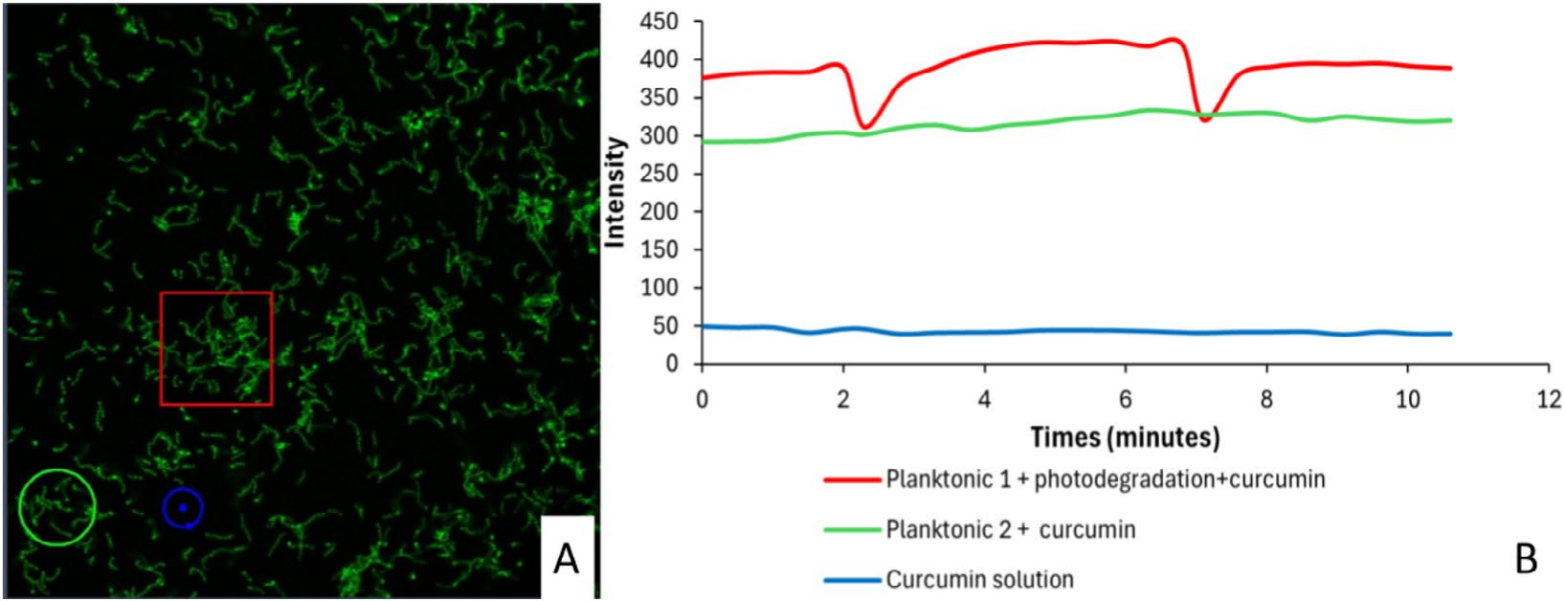
(A) Confocal microscopy image of *S. mutans* planktonic cells showing three selected areas for analysis: Area 4 (red square) represents planktonic cells subjected to photodegradation and curcumin treatment; Area 5 (green circle) represents planktonic cells treated only with curcumin without photodegradation; Area 6 (blue circle) represents the curcumin solution in the medium. (B) Graph showing intensity changes over 10 min. Planktonic 1 (red line) demonstrates a decrease in intensity following photodegradation and subsequent recovery, while Planktonic 2 (green line) shows stable intensity with only curcumin treatment. The curcumin solution (blue line) in the medium remains constant, indicating no photodegradation. Vertical black bars indicate the timing of photodegradation events. 20 μm scale.

Subsequently, the above results were compared with the planktonic forms (Figure 6). Planktonic cell 1 area (red rectangle, number 4) represents planktonic bacteria treated with curcumin and exposed to light (photodegradation). It presented the highest initial intensity and variations over time—recovering in around 1 min—indicating the influence of light on the cells in which the photosensitizer was internalized and a faster recovery time compared to the biofilm. Planktonic cell 2 (green circle, number 5) represents planktonic cells treated only with curcumin, without exposure to light. It maintained a more stable and lower intensity than the light‐treated group, suggesting the fluorescence of curcumin remains relatively constant in the absence of photodegradation.

**FIGURE 7:**
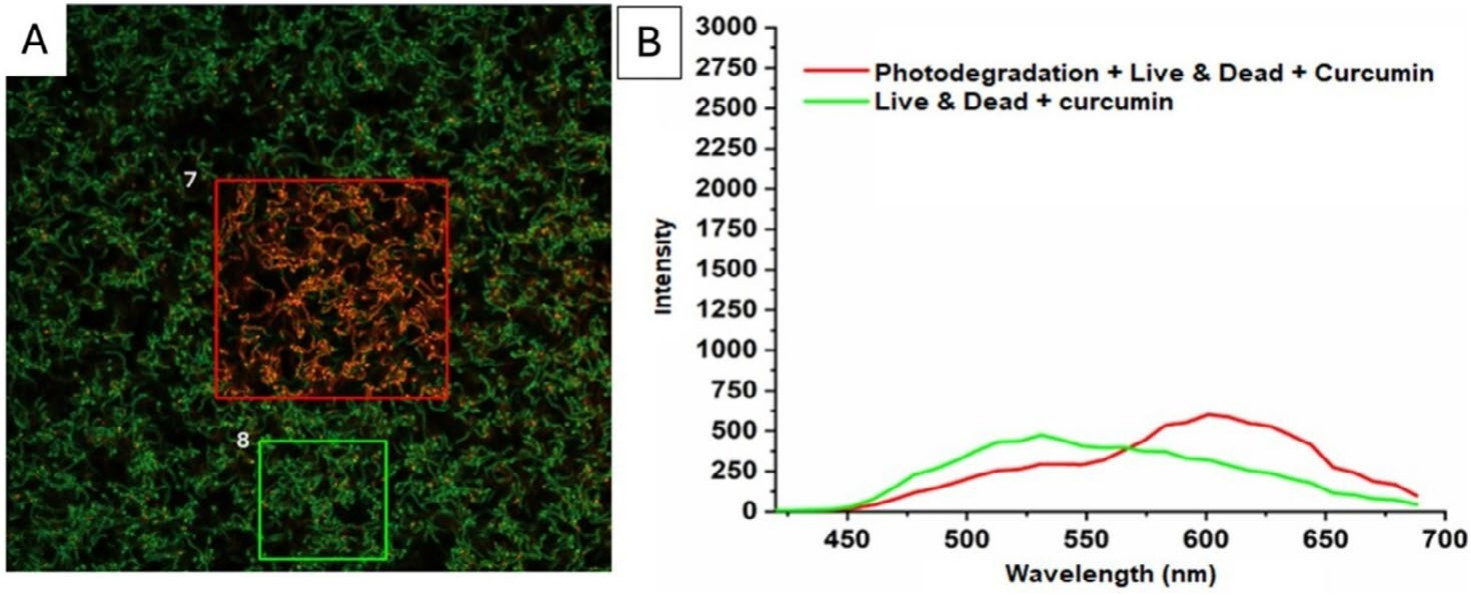
(A) Confocal microscopy image of *S. mutans* biofilm showing two areas: Area 7 (red square) represents biofilm subjected to photodegradation, Live & Dead staining, and curcumin treatment; Area 8 (green square) represents biofilm treated with Live & Dead staining and curcumin, without photodegradation. (B) Fluorescence intensity spectrum across wavelengths (450–700 nm) showing the emission profile of biofilms. The red line represents the intensity from the biofilm after photodegradation combined with Live & Dead staining and curcumin treatment, while the green line corresponds to the biofilm treated only with Live & Dead staining and curcumin. Photodegraded areas exhibit higher intensity in the red region, indicating an increase in cell death. 20 μm scale.

The results of the control in Figure 7 show the effects of photodegradation on samples treated with photosensitizer and Live & Dead dye. Figure 7A shows bacterial cell unviability, stained red, in the selected area (red square, number 7) where photodegradation was applied and another area (green square, number 8) with viable bacteria stained green. In addition, in Figure 7B, there is a fluorescence spectrum (Intensity × Wavelength–nm) which shows a higher fluorescence peak in the Photodegradation + Live & Dead + Curcumin group (red line), mainly in the region above 550 nm. This suggests light has caused changes in internalized curcumin and biofilm structure, resulting in greater fluorescence emission and corroborating Live & Dead's qualitative result of cell unviability in these bacteria. Also, in Live & Dead + curcumin without light (green line), fluorescence intensity is lower compared to the red line, and the spectrum appears slightly shifted. This indicates that in the absence of light, curcumin does not degrade significantly, and its fluorescence remains relatively stable.

**FIGURE 8:**
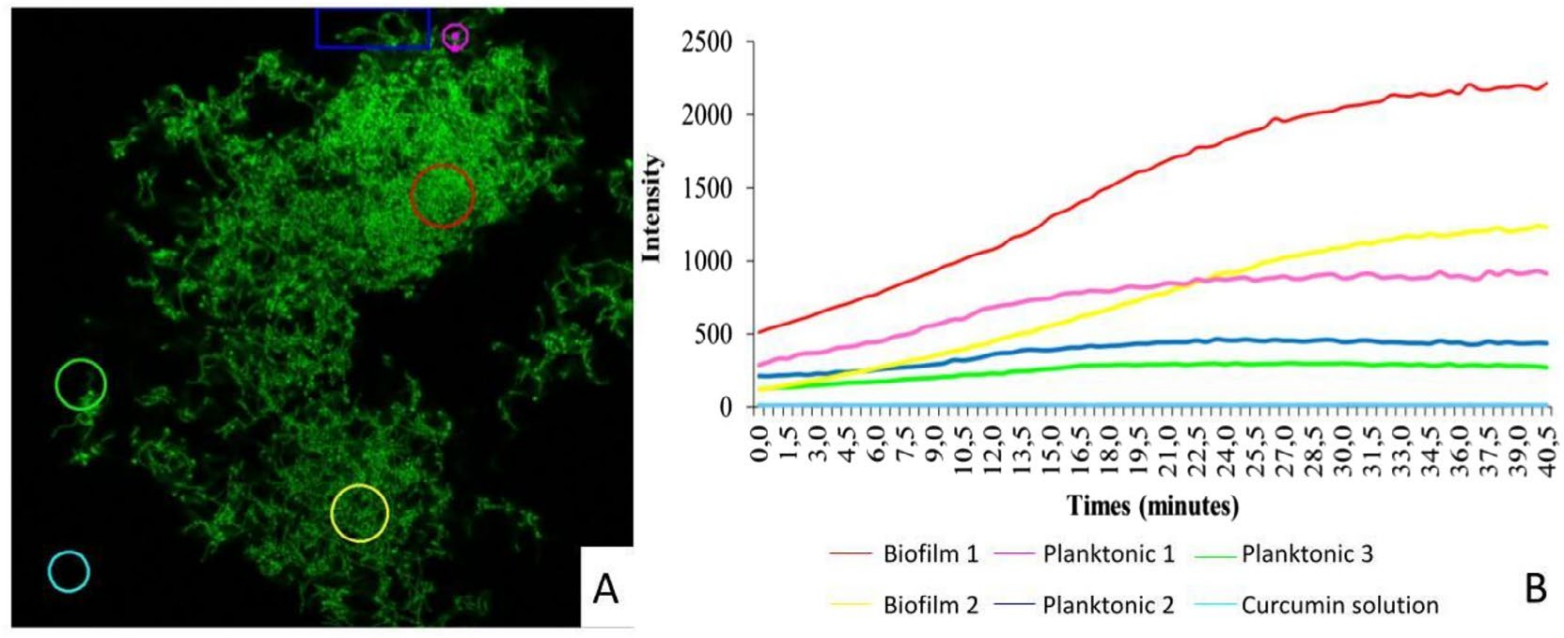
(A) Confocal microscopy image showing selected regions of *S. mutans* biofilm (red and yellow circles) and planktonic forms (pink circle, blue square and green circle) analyzed for fluorescence intensity. (B) Graph showing fluorescence intensity changes over 40 min. Biofilm 1 (red line) and Biofilm 2 (yellow line) show a continuous increase in fluorescence intensity, indicating higher curcumin internalization compared to planktonic samples. Planktonic 1 (purple line), Planktonic 2 (blue line), and Planktonic 3 (green line) exhibit slower intensity increases, highlighting the difference in curcumin uptake between biofilm and planktonic forms. The curcumin solution (cyan line) remains constant throughout the experiment. 20 μm scale.

Areas were randomly outlined on the sample in the same confocal plane (Figure 8A) to investigate curcumin uptake in biofilm and planktonic forms. According to the spectrum (Figure 8B), biofilm (red and yellow lines) has a higher capacity to internalize curcumin compared to planktonic cells (pink, blue and green lines). This indicates differences in permeability to photosensitizer uptake due to the extracellular composition of the bacteria in the biofilm. When comparing curcumin uptake, planktonic cells reached saturation earlier due to their intense metabolic activity, with curcumin uptake remaining constant after 10 min, while the uptake intensity in biofilm increased approximately seven times more than in planktonic form.

## 4 Discussion

Biofilm formation is a strategy employed by pathogenic bacteria to establish localized infections. Consequently, the issue of bacterial resistance and treatment difficulty becomes even more problematic [26, 27, 28]. The three‐dimensional structure of the biofilm functions as a microenvironment supported by a self‐secreted matrix, ensuring the survival of microorganisms [29]. Additionally, microorganisms within the biofilm are substantially more resistant to antimicrobial agents than their planktonic counterparts [29, 30, 31]. This resistance is partly due to the difficulty antibiotics face in penetrating biofilms [32, 33], as well as the challenges encountered by negatively charged PS with low water solubility in permeating these structures. Furthermore, the need to adjust the wavelength and dosage of the applied light complicates the PDI process [34, 35].

Accordingly, a preliminary literature review was performed to determine the average emission wavelengths used in fluorescence techniques involving curcumin‐conjugated biological systems, particularly within the 500–700 nm range [36]. Additionally, excitation and emission filters were adjusted to minimize interference from the excitation source, resulting in an applied wavelength in the 800 nm range. Accordingly, the results of this study contribute significantly to the understanding of curcumin‐mediated PDI in *S. mutans* biofilms. By demonstrating that curcumin internalization occurs more slowly and efficiently in biofilms than in planktonic forms, and that the recovery time after photodegradation is faster in planktonic forms than in biofilms, this study highlights the biofilm as a significant physiological barrier that can be overcome with the use of an appropriate photosensitizer. Previous studies have suggested that natural PS face limitations in treating biofilms [20], but our findings show that curcumin, when combined with blue light, exhibits efficient fluorescence recovery after photodegradation, indicating a high penetration rate and replacement of degraded curcumin. Compared to previous studies, our findings highlight the enhanced penetration capacity of curcumin in biofilms, suggesting its improved potential for photodynamic therapy [37].

A further limitation of this study is the use of single‐species biofilms, which do not fully represent the complexity of polymicrobial communities typically found in clinical infections. In real infections, biofilms often consist of multiple species of microorganisms. A recent study shows different responses in pure and mixed bacterial cultures after PDI treatment, indicating that further studies should be developed, as mixed biofilms may also exhibit distinct behaviors [38].

Although our results indicate that curcumin is promising, future studies should focus on investigating the optimal concentrations of curcumin to maximize photoinactivation across different types of biofilms. Additionally, exploring the combination of curcumin with other PS or antimicrobial agents to assess potential synergistic effects would be valuable. In vivo testing is also crucial to validate the clinical efficacy of curcumin in more complex environments, such as multispecies oral biofilms. Another avenue of investigation could involve encapsulating curcumin in nanoparticles to enhance its stability and delivery to target cells, thereby maximizing its effectiveness in photoinactivation.

In conclusion, adjusting the components necessary for effective photodynamic action on oral biofilms reinforces the potential of photodynamic therapy in reducing microorganisms present in biofilm‐related infections. These findings suggest that photodynamic therapy holds promise as a treatment for oral infections [39, 40, 41, 42, 43].

## Author Contributions

**Rebeca Vieira de Lima:** validity testing, writing – original draft preparation, visualization, investigation, reviewing and editing. **Bruno Pereira de Oliveira:** conceptualization, validity testing, drafting figures, supervision, reviewing and editing. **Francisco Eduardo Gontijo Guimarães:** supervision, reviewing and editing, data curation, validity testing. **Kate Cristina Blanco:** supervision, reviewing and editing, and validity testing. **Vanderlei Salvador Bagnato:** methodological supervision, analysis of the data, and interpretation of the results, financial resources.

## Conflicts of Interest

The authors declare no conflicts of interest.

## Data Availability

The data that support the findings of this study are available from the corresponding author upon reasonable request.
